# Self-assembling protein nanoparticles in the design of vaccines

**DOI:** 10.1016/j.csbj.2015.11.001

**Published:** 2015-11-26

**Authors:** Jacinto López-Sagaseta, Enrico Malito, Rino Rappuoli, Matthew J. Bottomley

**Affiliations:** GlaxoSmithKline Vaccines S.r.l., Via Fiorentina 1, 53100 Siena, Italy

**Keywords:** Virus-like particle (VLP), Ferritin, Lumazine synthase, HBV, HCV, HIV, HPV, Malaria, Influenza, Structural biology

## Abstract

For over 100 years, vaccines have been one of the most effective medical interventions for reducing infectious disease, and are estimated to save millions of lives globally each year. Nevertheless, many diseases are not yet preventable by vaccination. This large unmet medical need demands further research and the development of novel vaccines with high efficacy and safety. Compared to the 19th and early 20th century vaccines that were made of killed, inactivated, or live-attenuated pathogens, modern vaccines containing isolated, highly purified antigenic protein subunits are safer but tend to induce lower levels of protective immunity. One strategy to overcome the latter is to design antigen nanoparticles: assemblies of polypeptides that present multiple copies of subunit antigens in well-ordered arrays with defined orientations that can potentially mimic the repetitiveness, geometry, size, and shape of the natural host-pathogen surface interactions. Such nanoparticles offer a collective strength of multiple binding sites (avidity) and can provide improved antigen stability and immunogenicity. Several exciting advances have emerged lately, including preclinical evidence that this strategy may be applicable for the development of innovative new vaccines, for example, protecting against influenza, human immunodeficiency virus, and respiratory syncytial virus. Here, we provide a concise review of a critical selection of data that demonstrate the potential of this field. In addition, we highlight how the use of self-assembling protein nanoparticles can be effectively combined with the emerging discipline of structural vaccinology for maximum impact in the rational design of vaccine antigens.

## 1 Introduction

Vaccines are among the most outstanding achievements in human medical history. Through their power to prevent or reduce the burden of infectious diseases they make an enormous global impact by improving the life quality of both humans and animals. Vaccines may save up to three million children’s lives and up to six million total lives each year [1], [2]. In addition to their contribution to an increased survival rate, vaccines are also an essential medical tool to protect against cancers and devastating sequelae derived from viral and bacterial infections, such as human papillomavirus (HPV) or meningitis, allergies, autoimmune diseases, or even drug dependencies.

However, there are many important pathogens against which vaccines do not yet exist, and some current vaccines could be improved. For example, some vaccines do not protect against all circulating strains of a pathogen because many microbes have developed sophisticated mechanisms to escape the host immune system. Mutations on the antigens of microbes such as influenza (flu), human immunodeficiency virus (HIV), or meningococcus constitute a rapidly changing ‘disguise’ to avoid recognition by trained immune cells that might otherwise prevent infection or disease. Further, some vaccine antigens do not elicit sufficiently durable or potent immunity. In addition to this, the rise of drug-resistant pathogenic entities such as those causing shigellosis demands our attention in the search for proficient vaccines [3]. Therefore, a major research focus is to seek ways to boost vaccine-induced host protection against pathogens, by developing novel antigens that evoke a more robust and protective immune response.

Many effective vaccines developed in the past used live-attenuated strains of a pathogen, or inactivated killed pathogens [4]. Live-attenuated vaccine strains are typically highly immunogenic, but carry inherent safety concerns, given the potential of these weakened viral particles to revert into disease-causing viruses. Additionally, mutagenic events within the host organism may generate more virulent strains. Conversely, while inactivated or killed vaccine pathogens cannot replicate nor revert into more virulent forms, they tend to stimulate a weaker immune reaction, and thus may require the administration of multiple dosages, an important practical limitation. An effective way to address these limitations has gradually emerged through studies of self-assembling proteins, which can be used as nanoparticles mediating multi-copy antigen display.

One of the earliest examples of a self-associating protein particle was reported in the 1950s: a protein extracted from the tobacco mosaic virus (TMV) was found to form rod-shaped particles, which morphologically resembled the original TMV but which did not contain genetic material [5]. Later, in the 1970s, the hepatitis B virus (HBV) surface antigen (HBsAg) was purified from infected human serum [6]. Electron microscopy (EM) and ultraviolet absorption studies revealed that HBsAg formed spherical particles with an average diameter of ~ 22 nm and which, importantly, like the TMV protein particle, lacked nucleic acid and hence were non-infectious. Preparations of such virus-derived ‘nanoparticles’ formed the first efficacious HBV vaccine, licensed in 1981 [7], and represented a milestone that created a new focus in the field of vaccinology. Indeed, antigen nanoparticles, first exemplified by HBsAg, have now emerged as a leading strategy in the development of safe and potent vaccines.

**Fig. 1:**
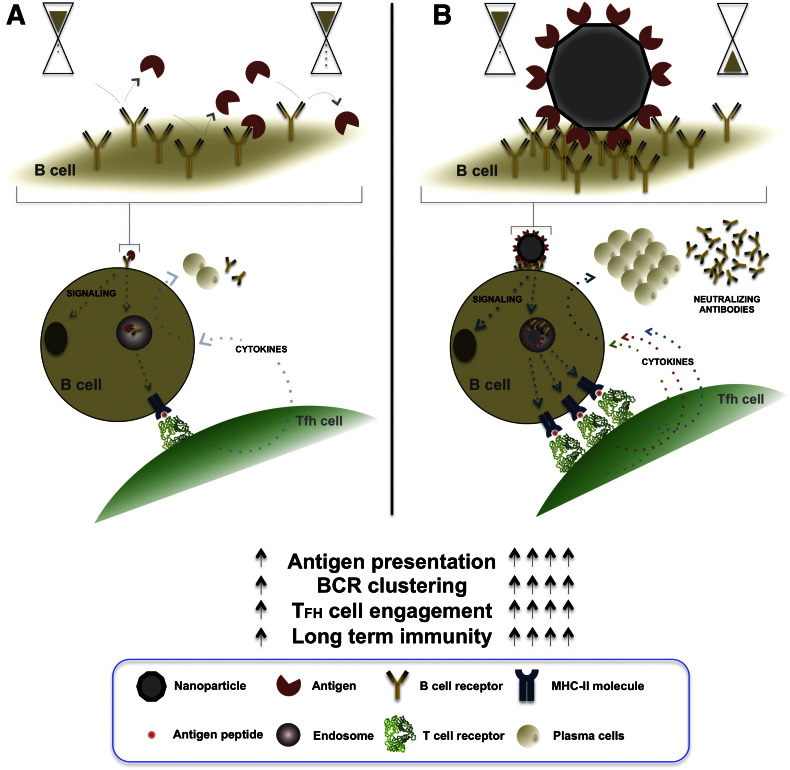
Multivalent nanoparticles favor the generation of potent, long-lived immunoprotection in germinal centers. Recombinant nanoparticles loaded with the desired antigen are designed thoroughly to present multiple copies of a pathogen epitope in a highly ordered manner on the surface of a self-assembling nanoparticle. As opposed to single recombinant antigens that provide brief half-life 1:1 interactions with the BCRs (A), the polydentate nature, i.e. avidity, of the interaction with the nanoparticle enables tighter and prolonged bindings: the dissociation of one antigen molecule can be compensated by the binding of a new antigen molecule or re-association with a new BCR (B). This scenario enables the clustering of BCRs for multiple and simultaneous engagement with the antigen epitopes. Thus, the B-cell traps the antigen-loaded nanoparticle to establish a durable, localized and strong recognition that translates into B-cell intracellular signaling, internalization and processing of the antigen for presentation, via molecules of the MHC complex, to the T follicular helper cells (Tfh) within the germinal centers. This new recognition evokes the secretion of regulatory cytokines by the Tfh cell and ultimately the evolution of B cells into plasma cells that can secrete antigen-specific neutralizing Abs.

What are the advantages of nanoparticle antigens? Key parameters governing the elicitation of an efficient immune response to a microbe include both antigen density and distribution on the pathogen surface [8]. B- and T-cell stimulation and activation, and the subsequent secretion of antigen-specific antibodies by plasma cells, rely on effective cross-linking between B-cell surface immunoglobulins (the B-cell receptor, BCR) and the recognition pattern presented by the pathogen. The high density and structurally ordered antigenic array presented by a nanoparticle provides a molecular scenario where multiple binding events occur simultaneously between the nanoparticle and the host cell BCRs (Fig. 1). This multivalent molecular and cellular setting favors the fruitful network of stimulatory interactions, as opposed to the weaker effect of monovalent binding afforded by single soluble recombinant antigens. Indeed, the high avidity for the nanoparticle provided by the multivalent interaction constitutes a critical step in the induction of a potent immune reaction (Fig. 1). Because of these advantageous immunological and physicochemical properties, and their suitability for large-scale manufacturing via *Escherichia coli* (*E. coli*) or eukaryotic systems, nanoparticles are at the frontline of new vaccine therapeutics.

A variety of naturally occurring proteins can self-assemble into nanoparticles that are highly symmetric, stable, and structurally organized, with diameters of 10–150 nm [9], [10], a highly suitable size range for optimal interactions with various cells of the immune system [8], [11]. These nanoparticles normally play diverse physiological roles, but are of particular interest in the context of vaccine design because they can be used as self-assembling platforms for the display of an arranged and well-ordered matrix of a particular immunogen, thereby mimicking the repetitive surface architecture of a natural microbe, e.g. a spherical virus capsid [12].

While many natural proteins have acquired self-assembling properties during evolution [13], the *de novo* engineering of protein assemblies is challenging. Early proposals to rationally perform atomic and molecular manipulations to generate interesting new materials were mentioned by Richard Feynman in 1960 [14], [15]. Later, the idea of combining proteins as building blocks into higher-order structures via self-assembly was advanced [15], [16]. More recently, the feasibility of designing self-assembling proteins has improved via new computational approaches [17]. Consequently, using natural or engineered protein nanoparticle scaffolds, vaccinologists can now aim to add heterologous epitopes or antigens onto the ‘plain’ nanoparticle, thus representing a limitless source of possible ‘chimeric’ nanoparticle antigens. Such chimeric nanoparticles can be obtained by self-assembly, or by covalent chemical attachment of an antigen to a nanoparticle.

Since the emergence of nanoparticle vaccine antigens in the 1970s, numerous attempts to generate plain or chimeric nanoparticles with scaffolds from many origins have been described. Some of these are virus-like particles (VLPs) composed of single or multiple viral antigens, in some cases anchored in a lipid bilayer. Structural proteins from different microorganisms have served as templates for the production of such nanoparticles and for the presentation of immunogenic epitopes: the protein pIII of the filamentous phage f1 [18], the Ty component from *Saccharomyces cerevisiae*[19], the surface and core antigens of the hepatitis B virus [20], [21], surface or coat proteins of bluetongue virus [22], human parvovirus B19 [23], tobacco mosaic virus [24], the Picornaviridae virus [25], Sindbis virus [26], and papillomavirus [27], [28] are just some examples. This mini-review will focus on a subset of such studies. We also aim to draw the reader’s attention to how we believe the design of future candidate antigens can be optimized by combining structural vaccinology and nanoparticle research. Structural vaccinology is an emerging discipline that uses insights from structural and computational biology studies with neighboring fields such as formulation science, immunology, animal studies, and serology in order to design, evaluate, optimize, and deliver leading candidate vaccine antigens [29], [30]. As a minor note, synthetic nanoparticles made from non-polypeptide polymers, metals, or other solid supports, and the use of encapsulating particles as vaccine delivery systems are beyond the scope of the current review, and the reader is directed to alternative sources [31], [32], [33].

## 2 Biochemistry and applications of bionanoparticle antigens

### 2.1 Viral proteins and virus-like particles (VLPs)

Many viruses encode proteins that form stable nanoparticle structures, which self-assemble in infected host cells in order to package the viral genomes as a pre-requisite for propagation. However, if scaffold proteins are assembled in the absence of genetic material, then non-infectious non-replicating virus-like particles (VLPs) that closely resemble intact virions can be obtained. Consequently, viral nanoparticles or VLPs composed of one, or a few, recombinant self-assembling proteins were among the first antigen-nanoparticle candidates, some of which have indeed been developed into successfully marketed vaccines [4]. Indeed, the earliest VLPs were relatively simply obtained by self-assembly of single recombinant HBV or HPV capsid proteins. After excellent results obtained with these first ‘plain’ VLPs, several ‘chimeric’ VLPs were explored as platforms for the display of heterologous epitopes or antigens. Here, notable examples from both categories are discussed. We will also briefly discuss the more complex VLPs representing enveloped viruses. In contrast to VLPs made from highly purified protein capsids, enveloped VLPs (virosomes) are assembled by budding from the host cell membrane. More extensive reviews of viral or VLP vaccines, with a focus on the challenges of their industrial development and manufacturing, can be found elsewhere [34], [35].

In the second half of the 20th century, the first genetically engineered vaccine was developed by cloning the VP_3_ capsid protein of the foot-and-mouth disease virus (FMDV), which was demonstrated to be a safe, stable and effective polypeptide vaccine for cattle and swine [36]. The breakthrough of the genetic FMDV animal vaccine, coupled with the discovery of the HBsAg vaccine antigen purified from human serum, stimulated the search for a safe, genetic *in vitro*-produced vaccine to protect humans against HBV. In 1986, licensure was obtained for the first human vaccine based on a recombinant protein subunit antigen (HBsAg), which self-assembles into nanoparticles [37], [38]. Although these assemblies form nanoparticles, they do not very closely resemble the intact HBV virion and thus are not fully considered VLPs. Nevertheless, recombinant HBsAg nanoparticles are included in two safe and efficacious vaccines (marketed as Engerix-B [39] and Recombivax-HB), now globally implemented to protect against HBV.

Perhaps the first real VLP human vaccine is the successful story of two similar vaccines protecting against HPV infection, a major cause of anogenital disease and, especially, cervical cancer [40]. Both vaccines were launched in the first decade of the 21st century (marketed as Cervarix and Gardasil) and contain recombinant HPV L1 major capsid protein VLPs [27], [28]. The development of these vaccines followed work in the 1980s, when the first reports emerged describing the self-assembly of recombinant forms of the major capsid proteins of several viruses, including hepatitis B [37], polyoma [41], and parvovirus [42]. Studies in the early 1990s then revealed that recombinant L1 from bovine and human papillomaviruses could self-assemble into empty capsid-like nanoparticles of ~ 50 nm diameter and, importantly, L1 nanoparticles could raise high-titer neutralizing antibodies in animals, with an immunogenicity profile similar to that of infectious HPV virions [43], [44]. Notably, the denatured non-assembled form of L1 did not induce neutralizing antibodies [40], highlighting the importance of the correct nanoparticulate structure of the protective epitopes in the L1 VLPs.

Several high-resolution crystal structures of different HPV L1 proteins have been determined, revealing the molecular basis for their oligomerization into the pentameric assembly unit of the viral shell [45]. Further, cryoEM studies have enabled reconstructions of the entire HPV type 16 capsid alone or bound to neutralizing Fab fragments [46], [47], [48]. Together, these structural studies have provided a deep understanding of the HPV L1 structure, antigenic specificity, and assembly into nanoparticles. Such L1 VLPs derived from HPV types 16 and 18 (in the bivalent Cervarix) and additionally types 6, 11 (in the first quadrivalent Gardasil), 31, 33, 45, 52, and 58 (in the nonavalent Gardasil 9) form the basis of safe and highly efficacious vaccines [49] (Table 1). Both HPV vaccines currently have relatively high production costs and their strain coverage efficacy is specific only for these L1 types included in the formulation, thus leaving room for improvement of potential second-generation vaccines. Nevertheless, the HPV L1 VLPs represent a shining example that encouraged the development and implementation of additional nanoparticle vaccines.

In addition to the research and clinical development of the HPV nanoparticle vaccines, numerous other viruses (for example, rotavirus [50], poliovirus [51], herpesvirus [52], and parvovirus [53]) have been used to generate non-infectious VLPs as vaccine candidates, although currently mostly without successful clinical development. However, the increased understanding of the self-assembly of these nanoparticles and VLP production technology led to new opportunities in making chimeric VLPs that display heterologous epitopes or antigens attached to the VLP either by covalent modification (chemical cross-linking) or through genetic engineering, discussed further below.

The first viral nanoparticles to be discovered, efficiently produced recombinantly and characterized were those from HBV, composed either of the surface antigen (HBsAg) [37], as described above, or the core antigen (HBcAg) [54]. The HBcAg was first shown to self-assemble into particles of 24–31 nm diameter, which resembled the viral cores obtained from HBV-infected human liver, and which were highly immunogenic in animals. Later cryoEM studies revealed that HBcAg produced in *E. coli* self-assembles into two classes of differently sized nanoparticles of 300 Å and 360 Å diameter, corresponding to 180 or 240 protomers [55], [56]. A landmark study by Brown and co-workers showed that a chimeric recombinant form of HBcAg genetically fused to the foreign FMDV peptide epitope could be produced in a viral expression system and self-assembled into nanoparticles displaying the FMDV epitope. This chimera was significantly more potent than the free FMDV peptide or the same peptide coupled to a beta-galactosidase carrier protein, and was highly potent in raising neutralizing antibodies against both FMDV and HBV [20]. Many analogous studies were subsequently performed, for example, showing that a human rhinovirus peptide presented on the HBcAg particle was 100-fold more immunogenic than uncoupled peptide [57]. Overall, many such studies revealed a number of sites on HBV nanoparticles suitable for insertion and display of foreign epitopes, in order to ensure optimal presentation to the immune system. It also emerged that carrier-specific immunosuppression and pre-existing immunity to HBcAg did not significantly alter the immunogenicity of the chimeric particles, thus potentially allowing repeated immunizations with the same nanoparticle platform [58]. Collectively, these findings supported the use of HBcAg nanoparticles or similar VLPs as efficient scaffolds for the presentation of heterologous (‘foreign’) epitopes as vaccine antigens.

Indeed, in the long search for a vaccine against malaria, successful clinical trials have been performed using chimeric nanoparticle antigens containing epitopes from the circumsporozoite protein (CSP) of the *Plasmodium falciparum* (*P. falciparum*) malaria parasite genetically fused either to HBcAg [59] (in Malarivax) or to HBsAg (in Mosquirix) [60]. This latter construct, presenting CSP residues 207–395 of *P. falciparum* NF54, is the key component of the RTS,S (Mosquirix) vaccine for which in mid-2015 the European Medicines Agency expressed a positive scientific opinion, following large-scale safety and efficacy data from phase III clinical trials [61], [62], [63] (Table 1). Although the latter is probably the most notable and clinically advanced new application, the literature contains many other examples of other nanoparticles or VLPs used as carriers for epitopes, some of which have entered clinical trials. For example, the NicQβ vaccine, which presents hundreds of copies of a nicotine hapten covalently attached to a self-assembling nanoparticle made of the bacteriophage Qβ coat protein, was shown to be safe and able to generate antibody responses potentially beneficial for smoking cessation [64]. Similarly, Qβ entered clinical trials as a platform on which a peptide representing the Aβ_1–6_ epitope was conjugated for display as an immunotherapy against Alzheimer disease [65].

**Fig. 2:**
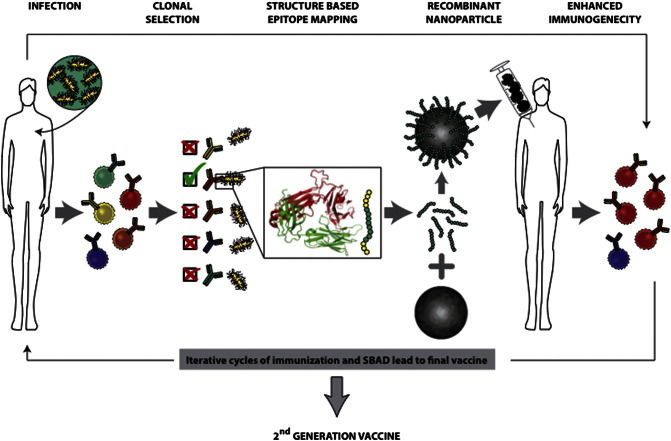
A flow diagram illustrating how human immunology, B-cell cloning, epitope mapping, structural vaccinology, and nanoparticle design can be combined in order to generate next-generation antigen-nanoparticle vaccines. Iterative cycles of structure-based antigen design (SBAD) can be performed to optimize the candidate antigens.

Interestingly, HBcAg was recently used in successful preclinical studies as a nanoparticle scaffold presenting a structurally optimized antigen against respiratory syncytial virus (RSV), a leading cause of severe respiratory tract disease in children worldwide [66]. Many previous efforts to design vaccine antigens to protect against RSV focused on the surface-exposed fusion glycoprotein, F, a highly conserved target of neutralizing antibodies [67]. Schief and co-workers performed a pioneering study by using insights from the crystal structure of a neutralizing epitope of the F antigen in order to computationally design and optimize its stabilized conformation for conjugation and presentation on HBcAg nanoparticles. The antigen alone was moderately immunogenic, but showed a much higher ability to induce protective RSV-neutralizing antibodies in several animal models including rhesus macaques when it was presented in multiple copies on the HBcAg scaffold [68]. For this formulation, the designed RSV F epitope was chemically conjugated to a form of HBcAg produced in *E. coli* and which exposes a reactive Lys genetically engineered into the major immunodominant region of HBcAg [69]. This bipartite approach of separately producing and purifying the optimized antigen and carrier components, followed by their conjugation, avoids the potential size limitation of antigens that can be incorporated into a nanoparticle via genetic fusion [70]. Although this production process was thus rather complicated, the neutralizing activity elicited was comparable to the titers induced by natural human infection, suggesting that this proof of principle for epitope-focused structure-based antigen design combined with self-assembling nanoparticle display holds great promise for future vaccines (Fig. 2).

Hepatitis E virus (HEV) constitutes another successful example of the generation of recombinant VLPs with efficacy in preventing the progress of the infection. While insect cells have been used to prepare HEV particles of a truncated version of the HEV viral capsid protein that yielded an antigenicity similar to that of original HEV viral particles [71], a bacteria-derived HEV particle with a shorter polypeptidic subunit rendered up to 86.8% efficacy and is currently in phase IV clinical trials (Hecolin) [72], [73].

The need for next-generation vaccines against influenza has been one of the biggest drivers of research into novel VLP antigens. For several decades, influenza vaccines have been successfully produced using embryonated chicken eggs [4]. Nevertheless, considerable efforts have been invested to devise alternative production methods for recombinant influenza antigens that might increase the vaccine manufacturing speed, yields, volume, purity and safety, and overcome the potential inability to provide sufficient vaccine doses in the event of future widespread epidemics or pandemics. In particular, several groups have explored influenza virus-like particles (VLPs): self-assemblies of the hemagglutinin (HA) and neuraminidase (NA) antigens on a lipid bilayer supported by the M1 matrix protein, generating non-infectious particles of approximately 100–150 nm diameter, thus resembling influenza virions [10], [74]. Following progress in making recombinant self-assembling VLPs from Sf9 insect cells [75], influenza VLPs were shown to be promising immunogens [76]. This recombinant system enables tailored antigen expression levels, overcoming the issues of low abundance of NA or M1 in the traditional egg-based influenza vaccines. Using this system, a recombinant VLP designed to protect against the avian influenza A H7N9 strain was generated [77]. The latter induced protective immunity in ferrets [78] and showed positive phase I clinical results [79]. Moreover, several similar VLP vaccines have been tested in preclinical studies; for example, H7N9 VLPs were produced in mammalian 293T cells, and raised high titers of neutralizing antibodies in mice [80]. Further, mice were broadly protected by a vaccine cocktail of VLPs displaying H1, H3, H5 and H7 hemagglutinin antigens [81]. These data suggest that the influenza VLP approach may be applicable as a rapid response to potentially pandemic strains and, moreover, they can also be used to prepare seasonal quadrivalent influenza vaccines (QIV), for which clinical trials are ongoing.

Nevertheless, a novel approach for the prevention of influenza in individuals aged 18 and older has been achieved with the development of Flublok, the first recombinant influenza vaccine to have been licensed. This trivalent vaccine contains recombinant HA proteins of three strains of seasonal influenza virus [82] and its higher antigen load has been reported to improve immunogenicity and efficacy [83]. Produced in insect cells, these proteins adopt multimeric nanoparticulate structures of 20–40 nm in diameter as characterized by dynamic light scattering and electron microscopy.

While influenza is one of the most promising targets for recombinant VLP technology, other viral targets have proved more challenging, especially when several viral proteins are required for VLP assembly [34], [35]. Although the currently licensed rotavirus (RV) vaccines are very efficacious, improved RV vaccines are needed to provide greater protection in developing countries and to improve protection against mild gastroenteritis. To this end, several rotavirus VLPs have been tested in preclinical models. For example, double-layered RV VLPs (~ 60 nm diameter, obtained using insect cell expression) made of recombinant VP2 and VP6 proteins, the most abundant RV antigens, conferred partial protection in animals, but required adjuvant or priming with live-attenuated RV vaccine [84]. More recently, progress has been made using *E*. *coli* as a suitable low-cost scalable production system for VP2–VP6 rotavirus VLPs that elicited a strong antibody response and protection against RV-induced diarrhea in mice [85]. These RV studies suggest that such VLPs hold promise, but likely require additional optimization of composition, immunization route, and effective adjuvants to be efficacious in humans.

Attempts to make HIV vaccines have also included various different VLPs, mostly involving lipid-enveloped assemblies where the internal HIV Gag p24 provides support for exposure of trimers of Env, the crucial HIV target antigen for neutralizing antibodies. A number of preclinical studies have shown the potential of this approach to efficiently induce humoral and cellular immune responses [86]. The initiation of future human clinical trials using emerging HIV VLP vaccine candidates should be facilitated by the clinical development experience (manufacturing, safety, etc.) accrued in the generation of influenza and rotavirus vaccines described above.

Human norovirus (NoV) causes endemic viral diarrheal disease and may cause up to half of all gastroenteritis outbreaks worldwide, and therefore is gaining increasing attention as another pathogen causing a globally significant healthcare burden [87], [88]. Candidate NoV VLP vaccines have been developed using the recombinant VP1 protein (the major NoV antigen) which self-assembles into nanoparticles (diameter ~ 30 nm) closely resembling the native virion [89]. A number of clinical trials have been performed (Table 1) and showed that NoV VLP vaccines are well tolerated and can induce rapid, robust immune response in adults [88]. However, in the clinical trials reported to date, results demonstrate modest protection from infection and disease and some prevention of severe gastroenteritis in healthy subjects, while efficacy data for the key ‘at risk’ groups have not yet emerged [87]. While the VLP platform appears promising, a key question for NoV vaccine development is the breadth of protection against multiple variant genotype strains, potentially resolvable using multivalent NoV VLP cocktails.

The viral examples described above clearly demonstrate the success, and future promise, of using highly purified non-enveloped viral capsid proteins as plain or chimeric antigen nanoparticles. However, enveloped VLPs, due to the inclusion of a lipid bilayer in addition to the structural protein antigens, represent a more complex challenge that is now being partly overcome via breakthroughs in mammalian and insect cell expression technologies. Unlike the simpler non-enveloped viral protein nanoparticles, enveloped VLPs have more inherent safety issues due to potential contaminations from the expression system used, and may be more challenging to formulate with long-term stability profiles. Nevertheless, several enveloped VLP candidates are now in clinical trials.

### 2.2 Bacterial protein platforms

**Fig. 3:**
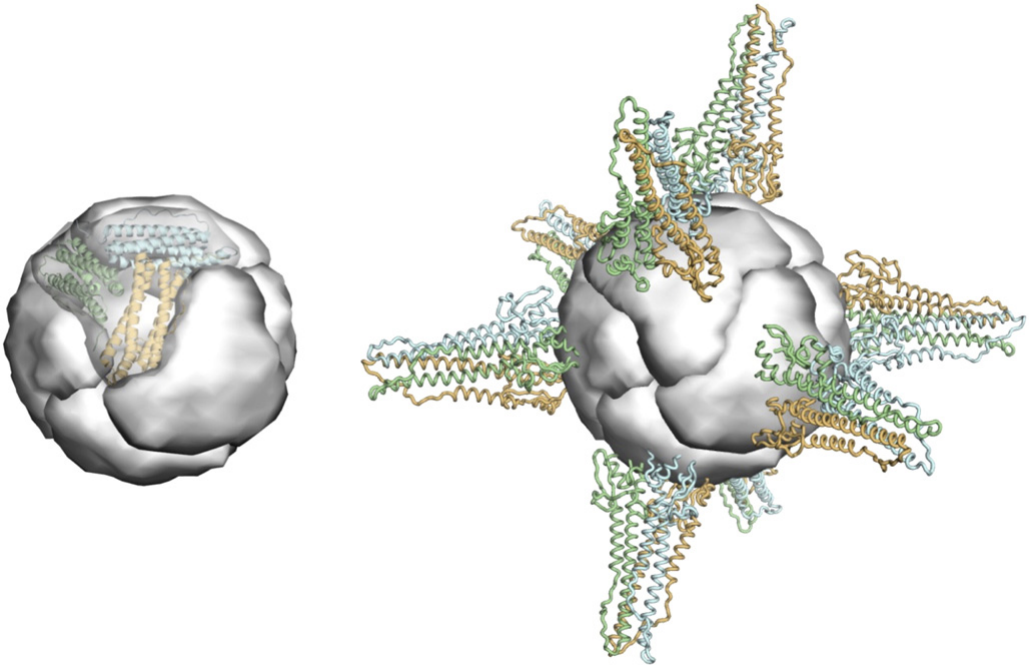
Generation of chimeric nanoparticles with surface-exposed arrays of immunogenic epitopes. Recombinant DNA technology can be used to make genes that encode self-assembling polypeptides fused with the desired immunogenic epitope for subsequent production in a chosen cell expression system. The chimeric polypeptide then self-assembles within the cell, with an ordered pattern of surface exposed epitopes. Here, we depict a model of ferritin shown as a grey isosurface (PDB: 3bve) that self-assembles in eight identical units, each composed of trimeric ferritin. On the left panel, one of the trimers can be visualized within one of the nanoparticle units. The monomers are colored in orange, green, and cyan. Given the intrinsic propensity of ferritin to self-assemble into a highly symmetric and ordered quaternary architecture, the chimeric nanoparticle is generated with the HA epitope (here shown on the right panel in orange, green, and cyan in cartoon-tube format (PDB: 3sm5)) incorporated and projected as a matrix of ordered and surface exposed epitopes ready for their recognition by BCRs, as described recently [93], [94]. The figure was prepared using Pymol software (The PyMOL Molecular Graphics System, Version 1.7.6.2, Schrödinger, LLC).

In addition to viral nanoparticles or VLPs, there are many other naturally occurring self-assembling protein nanoparticles that have been identified from a wide variety of sources [9]. For example, almost all living organisms produce ferritin, a protein whose main function is intracellular iron storage. Ferritin is made of 24 subunits, each composed of a four-alpha-helix bundle, that self-assemble in a quaternary structure with octahedral symmetry (Fig. 3). Several high-resolution structures of ferritin have been determined, confirming that *Helicobacter pylori* ferritin is made of 24 identical protomers [90], whereas in animals, there are ferritin light and heavy chains that can assemble alone or combine with different ratios into particles of 24 subunits [91], [92].

Ferritin self-assembles into nanoparticles with robust thermal and chemical stability. Hence, the ferritin nanoparticle is potentially well-suited to carry and expose immunogens. Moreover, since ferritin is composed of eight units each with three-fold axis symmetry, it is a convenient scaffold for the presentation of trimeric antigens. Indeed, Nabel and co-workers reported an elegant structure-based design strategy to generate ferritin nanoparticles genetically engineered to present a multivalent array of the flu virus hemagglutinin (HA) with its native trimeric conformation intact. HA is the key antigenic component of flu vaccines, and immunization of mice with these HA-ferritin nanoparticles yielded a very promising outcome: compared to a current commercial vaccine, the animals responded with a more potent immune response, as illustrated by the notably higher number of neutralizing antibodies, enhanced breadth of coverage against unmatched H1N1 viruses and increased generation of neutralizing Abs against two H1N1 highly conserved, yet independent, flu epitopes [93]. This work was one of the first clear examples of how the structurally optimized presentation of ordered arrays of a well-folded immunogen can induce stronger protection. In more recent work aiming towards a universal flu vaccine by providing broad coverage of protection against different subtypes of the flu virus, Yassine et al. reported a refined structure-based generation of a ferritin-based nanoparticle that displayed only the stem region of the H1 HA glycoprotein, yet was capable of evoking broadly cross-reactive antibodies that, in contrast to plain nanoparticles, protected mice and ferrets against lethal doses of heterosubtypic H5N1 virus [94].

Another similar study was also reported by the Nabel team, wherein the conserved receptor-binding domain (a site of vulnerability) of the gp350 antigen from Epstein–Barr virus was presented in a structurally optimized orientation on nanoparticles of ferritin (24 subunits) or encapsulin (60 subunits). In preclinical studies, the chimeric nanoparticle-gp350 antigens elicited 10- to 100-fold more potent virus-neutralizing antibody titers than the soluble gp350 antigens alone [95]. Importantly, in addition to their immuno-focusing ability to generate high-quality antibody responses, the recombinant nature of these nanoparticle antigens has the benefit of high purity, safety, and tolerability, further strengthening the appropriateness of this vaccination strategy (Table 2).

Also, very recent is the work by He et al., describing *in silico* studies to optimize molecular scaffolds for epitope presentation and leading to the generation of recombinant ferritin nanoparticles displaying epitope-scaffolds harboring E1 or E2 epitopes from hepatitis C virus, promising candidates for preclinical studies in the quest for an HCV vaccine [96]. This recent example applied to HCV builds on the epitope-scaffold rational design strategy that emerged in previous attempts to graft HIV epitopes onto heterologous protein scaffolds [97], and effectively combines this approach with the multivalent nanoparticle format.

Ferritin has also been used as antigen support in the search for potent and safe vaccine tools against HIV [98], which despite its identification more than 30 years ago remains as one of the most devastating pathogens afflicting the human population. In order to circumvent the fact that Abs others than the so-called broadly neutralizing antibodies (bNAbs) might occlude highly vulnerable HIV sites, Kwong and coworkers grafted a series of these HIV target motifs into different protein templates and the resultant chimeras were named ‘supersite transplants’. Transplants bearing a glycopeptide from the variable region 3 on gp120 were recognized by neutralizing antibodies from three different donors, and binding was enhanced by presentation of the transplants on ferritin nanoparticles.

Lumazine synthase (LS) represents another example of the inclusion of a bacterial particulate base for the optimization of vaccine candidates, as reported recently by Jardine et al. in their attempts to enhance the immunoreactivity of recombinant gp120 against HIV infection [99]. As mentioned before, HIV represents a major health problem worldwide. With 35 million people carrying the virus worldwide and a yearly morbidity of 1.7 million people (AVERT, http://www.avert.org/worldwide-hiv-aids-statistics.htm), the lack of a vaccine is an enormous unmet medical need. A key challenge in designing an anti-HIV vaccine is the high mutagenic capability of the virus and the unfeasibility of administering attenuated or killed virus because of safety issues. An additional hurdle is the negligible recognition potential of germline precursors of bNAbs, such as VRC01, against the wild-type gp120, the major immunogenic component of the HIV virus envelope. One way to overcome this obstacle was recently reported by Schief and co-workers [99], who boosted the affinity of the germline antibodies for the viral gp120 glycoprotein by displaying multiple copies of an engineered form of the antigen on a lumazine synthase (LS) nanoparticle.

LS, which is responsible for the penultimate catalytic step in the biosynthesis of riboflavin, is an enzyme present in a broad variety of organisms, including archaea, bacteria, fungi, plants, and eubacteria [100]. The LS monomer is 150 amino acids long, and consists of beta-sheets along with tandem alpha-helices flanking its sides. A number of different quaternary structures have been reported for LS, illustrating its morphological versatility: from homopentamers up to symmetrical assemblies of 12 pentamers forming capsids of 150 Å diameter. Even LS cages of more than 100 subunits have been described [101].

Using LS from the thermophilic bacterium *Aquifex aeolicus* as a nanoparticle platform for epitope display, Jardine et al. succeeded in increasing the potency of the immune response and breadth of coverage against HIV. The envelope (Env) glycoprotein is the only HIV surface protein targeted by neutralizing antibodies; it is made of three gp160 precursors that trimerize and are each then cleaved into gp120 and gp41 subunits. Jardine et al. engineered LS to display an optimized sub-component (termed, eOD-GT6) of the wild-type gp120 antigen from the Env trimer [99]. This approach overcame the issue that germline precursors of VRC01 bNAbs show undetectable affinity for wild-type Env. With additional structural stabilization of the trimer provided by an N-terminal coiled-coil GCN4 domain, the eOD-GT6 immunogen was fused to the C-terminus of the LS gene construct. The resulting recombinant nanoparticle antigens were efficiently obtained from mammalian cells, in stable and homogeneous self-assemblies of 60 LS monomers each presenting a glycosylated eOD-GT6. In contrast with the monomeric eOD-GT6 that did not stimulate B-cell activation, the LS-eOD-GT6 nanoparticles remarkably activated both germline and mature B cells. In accordance with related studies discussed above, Jardine et al. also hypothesize that the ability of the nanoparticles to induce cross-linking with the B-cell receptors was important to promote a successful immune response.

### 2.3 Micellar nanoparticles

A method to obtain protein micelles from full-length amphiphilic membrane proteins was developed and used to prepare viral surface proteins as water-soluble particles with a hydrophobic interior and a polar exterior, of relatively homogeneous size: approximately 20–30 nm diameter, depending on the protein [102]. Therein, Simons et al. predicted several possible applications of the approach, including the opportunity to make virus glycoprotein micelle vaccines. Indeed, a similar approach has been adapted for the preparation of protein nanoparticles comprised of amphiphilic antigens, where the protein micelles are prepared by extraction with non-ionic detergents from Sf9 insect cells expressing the recombinant antigen. In a compelling example, a slightly genetically modified full-length form of the RSV fusion (F) surface glycoprotein was extracted and purified from insect cell membranes and used to create protein nanoparticle micelles of ~ 40 nm diameter, where the trimeric F protein assembled into rosettes exposing conformational epitopes similar to those of the post-fusion F conformation and able to raise neutralizing Abs [103]. In very recent clinical trials, these RSV F antigen nanoparticles appeared safe, promoted immunogenicity, and reduced RSV infections [104], raising high expectations for a nanoparticle vaccine against RSV.

The micellar nanoparticle approach has also been exploited in the search for vaccines against the Coronaviridae virus (CoV) family, which represents an important group of emerging human pathogens, as witnessed in the severe acute respiratory syndrome SARS-CoV and Middle East respiratory syndrome MERS-CoV outbreaks of 2003 and 2012, respectively. Recombinant full-length forms of the major immunodominant CoV antigen—the amphiphilic spike glycoproteins—both from SARS-CoV and MERS-CoV were successfully obtained via non-ionic detergent-extraction from Sf9 cells. The purified spike proteins assembled into nanoparticles of ~ 25 nm diameter that, in adjuvanted formulations tested in mice, were capable of raising high-titer neutralizing antibody responses against the homologous virus [105]. These preclinical examples suggest that this protein nanoparticle approach may be suitable for rapid production of relatively simple but effective vaccines in response to emerging pathogens (Table 3).

### 2.4 New protein platforms

Proposals to perform molecular manipulations that, by exploiting chemical forces in a repetitious fashion, could lead to the production of interesting materials that date back at least to the 1960s [14], [15]. Indeed, in addition to the naturally occurring self-assembling proteins described above, several groups have explored ways to design and produce nanoparticle materials based on non-native polypeptides. For example, Burkhard and co-workers produced chimeric polypeptides capable of self-assembling into regular polyhedral nanoparticles [106]. The polypeptide consisted of an N-terminal pentamer-forming subunit derived from the cartilage oligomeric matrix protein (COMP), followed by a *de novo*-designed trimeric subunit domain. Both subunits present oligomeric coiled-coil conformations and importantly, the resultant synthetic molecule was shown to refold and self-assemble into nanoparticles with polyhedral symmetry. Alternative oligomerization motifs such as the trimeric foldon domain from fibritin have also been used in such designs [107]. The assembly of such nanoparticles gives rise to a multivalent molecular architecture that allows diverse immunogenic epitopes to be repeatedly displayed on the surface of the nanoparticles in a strictly arranged manner, a strategy that appears to be broadly applicable.

Indeed, using the polypeptide approach, the Burkhard team fused the C-terminal heptad repeat (HRC) region of the SARS-CoV spike protein in its pre-fusogenic state in frame with the nanoparticle scaffold [108]. This strategy allowed conservation of the trimeric coiled-coil conformation of the spike epitope. Immunization of mice with these SARS-nanoparticles successfully elicited neutralizing antibodies specific for the trimeric coiled-coil epitope of the pre-fusogenic HRC. Additional applications of this system targeted an HIV vaccine, by using a nanoparticle made of two covalently linked coiled-coil domains designed to incorporate the membrane proximal external region (MPER) of HIV-1 gp41 [109]. However, while high MPER-specific titers were raised by this nanoparticle, none of the sera displayed detectable neutralizing activity against HIV-1. More promisingly, similarly designed polypeptide nanoparticles displaying multiple copies of a rodent malaria epitope from the circumsporozoite protein of *Plasmodium berghei* elicited a long-lasting immune response [110]. Collectively, this preclinical research suggests that the self-assembling protein nanoparticle (SAPN) approach can generate safe non-native polypeptide antigens approximating the size and multivalent scenario of a virus and thus facilitate the recognition of the antigen by immune receptors.

Early in the 21st century, Yeates and co-workers developed the nanohedra protein-design method, which was subsequently extended by Noble and co-workers [112], [113]. The Yeates team rationally designed genetic fusions of the trimeric bromoperoxidase and the dimeric M1 matrix protein of influenza virus, such that the combination of the two naturally oligomeric proteins generated self-assembling nanostructures, including a 15-nm-wide molecular cage and a 4-nm-wide filamentous superstructure [112], [113]. Later, a well-ordered tetrahedral cage with 12 subunits was designed and its crystal structure was determined and revealed to closely match the intended design, validating this approach [113]. The Noble team used proteins with higher symmetry, allowing design of fusions with two or more connections, generating regular molecular arrays that formed protein lattices, but not closed nanohedral particles [114]. Several subsequent *in silico* and crystallographic studies have further developed nanostructure design strategies, including the generation of particles over 22 nm in diameter [115]. Therefore, with the development of these more powerful computational approaches for the *ab initio* design of new protein-protein interfaces with defined symmetry, geometry, and complementary packing arrangements, and the increasing number of protein structures in the PDB, it is speculated that additional achievements in the field of self-assembling protein design will be possible [96], [116], [117], [118], [119], [120], [121], [122], [123]. It will be interesting to see if such scaffolds can be fully exploited to display antigenic epitopes suitable for full development into clinically efficacious vaccines.

## 3 Conclusions

Despite many successes in the field of vaccinology, new breakthroughs are still needed to protect humans from several important life-threatening diseases. Here, we have reviewed how a variety of non-infectious biological nanoparticles can offer solutions. For example, some plain nanoparticles (e.g. HBsAg or the HPV L1 protein) are simple molecular self-assemblies that are safe and efficacious vaccine antigens licensed for human use. Or, more complex chimeric nanoparticles can be platforms on which pathogen-derived immunogenic motifs can be presented to the host immune system. These biological scaffolds range from synthetic polypeptides to native macromolecules such as ferritin, lumazine synthase or VLP-forming antigens or lipid-enveloped VLPs. For some chimeric nanoparticles, there is evidence that the immunogenicity of the platform carrier itself is negligible or low compared to that of the mounted immunogen being presented [93]. Because these nanoparticles display an ordered matrix of immunogens, they enable more fruitful engagements with the B-cell receptors than single recombinant immunogens can establish. Several animal models and clinical studies have been reported that exemplify the high efficiency of these nanostructures in eliciting potent and long-lasting immunity. Following successful clinical studies, a recombinant nanoparticle-based vaccine against malaria is emerging, and two vaccines are already available to prevent HPV-related diseases.

Recent preclinical studies have demonstrated how computational and structural biology can be combined for the rational design of well-oriented arrays of the most protective epitopes of a pathogen, in a manner suitable to raise the most desired immune responses. Further, the computational *ab initio* design of self-assembling molecules is becoming ever more possible, thus enriching our molecular repertoire of nanoparticle scaffolds. Consequently, the design of plain or chimeric nanoparticle antigens, and the ability to manufacture these recombinantly in prokaryotic or eukaryotic systems, to make safe and effective immunogens, is becoming a reality. We have shown that vaccination clinical trials against a broad range of diseases are ongoing. While there are established examples of successful vaccines against diseases of viral and parasitic origin (hepatitis B, HPV, malaria), the next decade may consolidate the use of multivalent nanoparticles as a therapeutic tool for the future against infectious diseases of other origins (bacterial, fungal) but also against cancers and other disorders such as hypertension, asthma, or addictions (e.g. smoking). These amazingly versatile tools may also get us closer to universal vaccines against highly variable pathogens such as HIV, influenza, or meningitis. Continued efforts are still needed, but there is a well-founded optimism that further studies of nanoparticles and VLPs together with the implementation of structural vaccinology, facilitated by emerging B-cell cloning and antibody production technologies, will be translated into new and second-generation vaccines that will contribute to saving more lives worldwide.

## Conflicts of interest

The authors are employees of GlaxoSmithKline Vaccines S.r.l., via Fiorentina 1, 53100 Siena, Italy.

## Figures and Tables

**Table 1:** A table listing nanoparticle platforms of viral nature, with their composition, production method and stage of (pre)clinical development.

Platform	Antigen	Target	Expression system	Stage	Ref.
HBsAg	HBsAg	Hepatitis B virus	Yeast	Several licenses	[36], [37]
HBsAg	*P*. *falciparum* CSP 207–395	Malaria	Yeast	Phase III	[53]
HBcAg	*P*. *falciparum* CSP T and B-cell epitopes	Malaria	Bacteria	Phase I	[58]
HBcAg	Glycoprotein F (fragment)	RSV	Bacteria + chemical conjugation	Preclinical	[67]
HBcAg	Influenza matrix protein 2	Influenza	Bacteria	Phase I	[78]
HEV	HEV capsid polypeptide	Hepatitis E virus	Bacteria	Phase IV	[79]
HPV L1	HPV L1 major capsid protein	HPV	Yeast	Several licenses	[26], [27]
Hemagglutinin	HA	Influenza	Insect cells	Licensed	[82]
Full length HA/NA/M1	HA, NA, M1	Influenza	Insect cells	Phase II	[78]
Bacteriophage Qβ	Nicotine hapten	Nicotine	Bacteria + chemical conjugation	Phase II	[63]
Bacteriophage Qβ	Aβ_1–6_ epitope	Alzheimer	Bacteria + chemical conjugation	Phase II	[64]
Bacteriophage Qβ	IL-1β	Type II diabetes mellitus	Bacteria + chemical conjugation	Phase I	[123]
Bacteriophage Qβ	Angiotensin II	Hypertension	Bacteria + chemical conjugation	Phase II	[124]
Bacteriophage Qβ	Peptide_16–35_ of MelanA/MART-1	Malignant melanoma	Bacteria + chemical conjugation	Phase II	[125]
Alfalfa mosaic virus	Peptides from rabies proteins G and N	Rabies	Plant	Phase I	[126]
NoV capsid protein	NoV capsid protein	Human norovirus	Insect cells	Phase I	[127]
NoV capsid protein	NoV capsid protein	Human norovirus	Plant	Phase I	[128]
Parvovirus B19 capsid proteins	Proteins VP1, VP2	Human parvovirus	Insect cells	Phase I	[129]

Abbreviations: HBsAg, hepatitis B surface antigen; HBcAg, hepatitis B core antigen; CSP, circumsporozoite; RSV, respiratory syncytial virus; HPV, human papillomavirus; HA, hemagglutinin; NA, neuraminidase; M1, matrix protein 1; NoV, norovirus.

**Table 2:** A table listing nanoparticle platforms of bacterial nature, with their composition, production method, and stage of (pre)clinical development.

Platform	Antigen	Target	Expression system	Stage	Ref.
Ferritin	GP350 CR2-binding domain	Epstein–Barr virus	Mammalian cells	Preclinical	[94]
Ferritin	E1/ E2 envelope proteins	Hepatitis C virus	Mammalian cells	Preclinical	[95]
Ferritin	Variable region 3 on gp120	HIV	Mammalian cells	Preclinical	[97]
Ferritin	HA	Influenza	Mammalian cells	Preclinical	[92]
Encapsulin	GP350 CR2-binding domain	Epstein–Barr virus	Mammalian cells	Preclinical	[94]
Lumazine Synthase	Engineered gp120	HIV	Mammalian cells	Preclinical	[98]

Abbreviations: HIV, human immunodeficiency virus; HA, hemagglutinin.

**Table 3:** A table listing nanoparticle platforms of diverse nature, with their composition, production method, and stage of (pre)clinical development.

Platform	Antigen	Target	Expression system	Stage	Ref.
Micellar protein	Glycoprotein F	RSV	Insect cells	Phase II	[103]
Micellar protein	Spike glycoprotein S	SARS and MERS coronavirus	Insect cells	Preclinical	[104]
Virosome	Her-2 peptides of Her-2/neu	Breast cancer	Cell free	Phase I	[130]
Virosome	Aspartyl proteinase-2	*Candida albicans*	Cell free	Phase I	[131]
Virosome	P1 and recombinant gp41	HIV	Cell free	Phase I	[132]
Virosome	HA, NA	Influenza	Cell free	One license	[133]
Ty p1	p17/p24	HIV	Yeast	Phase II	[134]
Synthetic polypeptide	Heptad repeat of CoV spike protein (pre-fusogenic state)	SARS	Bacteria	Preclinical	[107]
Synthetic polypeptide	Membrane proximal gp41	HIV	Bacteria	Preclinical	[108]
Synthetic polypeptide	*Plasmodium berghei* CSP B-cell epitope	Malaria	Bacteria	Preclinical	[109]

Abbreviations: CSP, circumsporozoite; RSV, respiratory syncytial virus; HA, hemagglutinin; NA, neuraminidase; SARS, severe acute respiratory syndrome; MERS, Middle East respiratory syndrome; HIV, human immunodeficiency virus.
